# Clinical Utility of Serum Neuropeptide Q in Patients With Acute Coronary Syndrome: A Case-Control Observational Pilot Study

**DOI:** 10.7759/cureus.117054

**Published:** 2026-09-28

**Authors:** A Shaik Mohamed Shameer, Prashant S Adole, Kolar V Vinod

**Affiliations:** 1 Department of Biochemistry, Jawaharlal Institute of Postgraduate Medical Education and Research, Puducherry, IND; 2 Department of General Medicine, Jawaharlal Institute of Postgraduate Medical Education and Research, Puducherry, IND

**Keywords:** acute coronary syndrome, diagnostic test, heart-type fatty acid binding protein, myocardial infarction, neuropeptide q

## Abstract

Objective: Current cardiac biomarkers lack specificity and are limited by instrumental constraints in diagnosing acute coronary syndrome (ACS). Therefore, research has focused on identifying new cardiac biomarkers, e.g., neuropeptide Q (NPQ), associated with the pathophysiological response to myocardial injury. NPQ plays an important role in metabolic regulation and cardiac protection. As there are limited studies on the role of NPQ in ACS, we assessed serum NPQ levels in ACS patients and healthy populations to evaluate serum NPQ’s clinical utility in ACS.

Materials and methods: We recruited ACS patients (n = 120) and non-hospitalized healthy participants (n = 40). The baseline characteristics and history were recorded. Biochemical investigations, including serum NPQ and heart-type fatty acid-binding protein (H-FABP), were measured using blood samples collected at a mean of 24 hours after the onset of chest pain in ACS patients and at the time of enrolment of healthy participants. Appropriate statistical tests were performed.

Results: ACS patients had significantly higher serum NPQ and H-FABP levels as compared to healthy participants. Serum NPQ levels showed a significant positive correlation with weight, waist circumference, creatine kinase-total, creatine kinase-MB, and H-FABP levels and showed a significant negative correlation with white blood cells (WBCs) and serum high-density lipoprotein-cholesterol (HDL-C) levels. In univariate logistic regression, weight, WBC, HDL-C, and NPQ levels were linked with ACS outcome in study participants. In multivariate logistic regression analysis, after adjusting for potential confounders, WBC and NPQ were independently linked with ACS outcome in the study population. Receiver operating characteristic curve evaluation showed that the cut-off value of serum NPQ level for prediction of ACS was 2.53 ng/mL, with a specificity of 67.5%, sensitivity of 71%, and an area under the curve of 0.808 (95% CI: 0.731-0.886).

Conclusion: The study showed that the reported performance characteristics of serum NPQ levels strictly differentiate between established ACS patients and apparently healthy participants. Its diagnostic utility when facing a standard chest-pain differential diagnosis remains completely unknown. However, our findings should be confirmed by further studies.

## Introduction

Cardiovascular disease (CVD), a medical condition that involves dysfunction of the heart and blood vessels, has become the leading cause of mortality and morbidity [1]. Globally, the prevalence of CVD increased from 311 million to 626 million, and the number of deaths due to CVD increased moderately from 13.1 to 19.2 million between 1990 and 2023 [2]. Aortic atherosclerosis, stroke, transient ischemic attack, coronary artery disease (CAD), and peripheral artery disease are all included in CVD. Among them, acute coronary syndrome (ACS) tends to be the most common form of heart disease. ACS is divided into two categories: ST-elevation ACS (STE-ACS), which primarily includes ST-elevation myocardial infarction (STEMI), and non-ST-elevation ACS (NSTE-ACS), which includes unstable angina (UA) and non-ST-elevation myocardial infarction (NSTEMI). The risk of ACS among Asian Indian populations was estimated to be three to four times higher than that of White American populations [3]. Currently, India has the highest burden of ACS, especially in the form of STEMI [4].

ACS patients typically present with acute chest discomfort, shortness of breath, and ST-segment elevation on electrocardiogram (ECG). However, in patients with no symptoms or ECG abnormalities, measuring cardiac biomarkers plays a significant role in diagnosing ACS. Traditional cardiac markers like serum creatine kinase-MB (CK-MB), natriuretic peptides (NPs), and cardiac troponins (cTnI and cTnT) were established parameters to diagnose ACS. Though they are routinely used, they have advantages and disadvantages in diagnosing ACS. Troponins are important in detecting heart damage; however, they are not specific to myocardial injury alone. Levels of troponins are elevated in myocarditis, pulmonary embolism, renal failure, etc. Also, delayed window, false positivity due to lab interferences, and over-sensitivity are problems associated with troponin estimations [5,6]. Research has been conducted to find an ideal and novel cardiac biomarker that will assist clinicians with risk assessment, early diagnosis, prognosis, and treatment of ACS [7,8].

Neuropeptide Q (NPQ), also known as spexin, is a peptide hormone with 14 amino acids. NPQ is expressed in various central and peripheral tissues, especially in the heart, and secreted into circulation in response to a variety of stimuli, such as metabolic stress. NPQ plays a multifunctional role in regulating body weight, food intake, glucose, and lipid metabolism. It decreases food intake and lowers body weight in animal models. It also improves glucose tolerance and insulin sensitivity, and reduces lipid accumulation in the liver [9]. Studies suggest that NPQ possesses an anti-inflammatory effect. It exerts its effect mainly by reducing pro-inflammatory cytokines and enhancing anti-inflammatory cytokines [10]. In addition to these, several experimental studies have demonstrated that NPQ has a cardioprotective effect. For example, administration of NPQ reduced dyslipidemia, improved insulin sensitivity, and protected cardiomyocytes from hypoxia-induced mitochondrial dysfunction and oxidative stress [11]. In humans, lower NPQ levels are linked with adverse lipid profiles, hypertension (HTN), obesity, and diabetes mellitus (DM) [12]. These findings suggest that NPQ may serve as a biomarker for ACS.

Due to the widespread presence and higher mortality rate in ACS patients, the need for ideal cardiac markers is more urgent for diagnosis, prognosis, and risk stratification. As NPQ has shown beneficial effects on cardiomyocytes and there is limited information about serum NPQ levels in ACS patients, the present study aimed to characterize serum NPQ levels in established ACS patients and to determine its prognostic value. The objectives of this study were to estimate and compare the serum levels of NPQ between patients with ACS and healthy individuals, to correlate these findings with classical clinical parameters and cardiac markers, and to assess the role of serum NPQ levels in risk stratification using Thrombolysis in Myocardial Infarction (TIMI), Global Registry of Acute Coronary Events (GRACE), and Killip scores [13-15]. Serum CK-MB, creatine kinase-total (CK-total), and heart-type fatty acid-binding protein (H-FABP) levels are used as cardiac markers for comparison with serum NPQ levels. Though serum H-FABP level is a marker for early diagnosis of ACS, its profiling post onset provides critical insights into ongoing myocardial injury and risk stratification.

## Materials and methods

Study type, location, duration, and ethical considerations

This case-control observational study was conducted by the Department of Biochemistry in collaboration with the Department of Medicine at Jawaharlal Institute of Postgraduate Medical Education and Research (JIPMER), Puducherry, India, from 2024 to 2025. This study was approved by the Departmental Postgraduate Research Monitoring Committee (PGRMC) on 01/02/2024 and the Institute Ethics Committee for Human Studies (JIP/IEC-OS/2024/10; dated: 06/04/2024). This study follows the World Medical Association Declaration of Helsinki’s ethical principles for medical research involving human participants. Written informed consent was obtained from all study participants or their relatives before participation.

Inclusion/exclusion criteria

Patients (over 18 years) with ACS, including STEMI, NSTEMI, and UA, were recruited as cases within 12-36 hours of the onset of chest pain or shortness of breath. STEMI is diagnosed by persistent ST-segment elevation or new left bundle branch block on ECG in patients with chest pain. NSTEMI is diagnosed with symptoms and a dynamic rise and/or fall of cardiac troponin above the 99th percentile upper reference limit, accompanied by at least one sign of ischemia (e.g., non-persistent ST-depression, T-wave inversion, or imaging evidence of a new regional wall motion abnormality). UA is diagnosed by myocardial ischemia presenting at rest or with minimal exertion in the absence of acute cardiomyocyte necrosis (i.e., normal or non-elevated serial cardiac troponin levels) [16]. Healthy participants (patient bystanders other than relatives/healthy volunteers/patients who were reported to the hospital for minor ailments) were recruited as controls and matched for age and gender with cases. Controls were screened by physical examination and blood chemistry panels. Patients with muscle disorders, bone diseases, symptomatic degenerative diseases, cancers, cardiac disease, chronic kidney disease (CKD) with serum creatinine level > 2.5 mg/dL, and symptomatic vascular diseases were excluded from the study.

Sampling technique and sample size calculation

Simple convenience sampling was adopted, with selection of cases from patients diagnosed with ACS presented in emergency medicine within 12 to 36 hours from the onset of chest pain (preferably 24 hours). Previous research regarding serum NPQ levels in ACS patients remains highly limited and conflicting. A recent study by Çiftçi et al. estimated serum NPQ levels in 100 acute myocardial infarction patients and showed that NPQ plays a quantifiable role in the pathophysiologic response to ischemic cardiac insult [17]. Therefore, the study was planned as a pilot study including 120 participants in cases and 40 in controls. Out of 40 controls, 35 were healthy participants, one was a patient bystander, and four were patients with minor ailments. A priori sample size calculation was not performed. To achieve the maximum statistical power, all eligible patients presenting to the casualty during the study period and who met the inclusion and exclusion criteria were enrolled (n = 120). Based on this, the primary area under the receiver operating characteristic curve (AUC) of 0.808 achieved a post-hoc 95% confidence interval of 0.731 to 0.886, representing an absolute margin of error of 0.08.

Data collection and study variables

General characteristics and personal and family history of medical illness were recorded. Anthropometric parameters and blood pressure were measured. Major adverse cardiac events (MACE) in patients with ACS were determined by TIMI and GRACE risk scores [13,14]. Seven elements were assessed in the TIMI score, and eight were assessed in the GRACE score [13,14]. ACS patients were stratified into four classes based on the Killip classification as per the severity of their post-MI heart failure [15,18]. Under aseptic precautions, 5 milliliters of venous blood from the cubital vein were collected from ACS patients at admission. The blood collection from healthy controls was done at the time of inclusion in the study. The serum was collected and stored at -40°C for a maximum of six months. NPQ was stored for six months at -40°C, with a maximum of three freeze-thaw cycles. Routine biochemical investigations like liver function tests, kidney function tests, cardiac biomarkers, random blood sugar, and lipid profiles were measured by a chemistry analyzer (AU5800, Beckman Coulter, Brea, CA) using appropriate methods. Hematological investigations were measured using a cell counter (XN-350TM, Sysmex America, Inc., Lincolnshire, IL). Serum NPQ levels were estimated using a Sandwich ELISA kit (E-EL-H5607, Elabscience, Houston, TX) with sensitivity of 0.047 ng/mL, detection range of 0.078-6.0 ng/mL, recovery of 80-100%, and intraday and interday precision of less than 10%. Serum H-FABP levels were estimated using a Sandwich ELISA kit (E-EL-H1431, Elabscience) with sensitivity of 0.1 ng/mL, detection range of 0.16-10.0 ng/mL, recovery of 80-120%, and intraday and interday precision of less than 10%. The quality control department of the manufacturer validated all enzyme-linked immunosorbent assay (ELISA) kits. The known concentrations (standards) of each analyte were analyzed, and the standard curve was prepared, which showed good linearity with R2 of more than 0.9954. The standards were run along with samples, which showed reproducibility of results. No sample dilution was done, and each sample was run in duplicate during the ELISA procedure.

Statistical analysis

The SPSS program (version 27.0, IBM Corp., Armonk, NY) was used for statistical analysis. The normality of data was assessed by the Shapiro-Wilk test. Continuous variables with a normal distribution were expressed as mean ± standard deviation (SD) and collated by the independent-samples Student’s t-test. Variables with a non-normal distribution were expressed as median (interquartile range) and collated by the Mann-Whitney test. Categorical variables were expressed as numbers (percentages) and collated by the chi-square test of homogeneity or Fisher’s exact test. The comparison of serum NPQ levels among more than two groups was analyzed using the Kruskal-Wallis test, whereas comparison between two groups was analyzed using the Mann-Whitney U test. Spearman’s and Pearson’s correlation coefficients were used to find the correlation of NPQ and H-FABP with clinical, biochemical, and patient characteristics. Due to the right-skewed distribution of H-FABP levels, log transformation was applied before regression analysis. Examination of residual histograms, normal probability plots, and residual scatter plots indicated that the assumption of linear regression was reasonably satisfied. Multiple linear regression was used to evaluate the association between serum NPQ and H-FABP levels after adjusting for weight, waist circumference, white blood cells (WBCs), and serum high-density lipoprotein-cholesterol (HDL-C) level, as they showed significant correlations with serum NPQ levels. Due to limited sample size within individual groups, subgroup analyses were conducted using grouped risk categories as follows: TIMI score was analyzed as low- and intermediate-risk groups, as no patients were present in the high-risk categories; GRACE risk scores were dichotomized into low-risk and intermediate/high risk groups; Killip classification was grouped as class 1 and class 2-4; and coronary angiographic findings were categorized as single-vessel disease (SVD) and combined double-vessel disease (DVD) and triple-vessel disease (TVD) for subgroup analysis. Univariate and multivariate binary logistic regression were performed to determine the predictive value (odds ratio and 95% confidence interval) of serum NPQ levels for ACS. We designated the initial receiver operating characteristic (ROC) analysis as the derivation phase to evaluate the predictive capability of serum NPQ and serum H-FABP levels for ACS. To evaluate and compare the discriminatory power of each parameter, we calculated the AUC-ROC. We identified optimal cut-off points using Youden’s Index. At these thresholds, we calculated the sensitivity, specificity, positive likelihood ratio (LR+), and negative likelihood ratio (LR-), along with their respective 95% confidence intervals (CIs). For all statistical tests, a p < 0.05 was considered statistically significant.

## Results

General characteristics and laboratory investigations

The initial demographics of the study participants are shown in Table 1.

**Table 1:** Clinical and biochemical properties of the study population. Data are expressed as median (interquartile range), mean ± standard deviation, or number (percentage), as appropriate. Normally distributed continuous variables were compared using Student’s t-test. Non-normally distributed variables were compared using the Mann-Whitney U test. Categorical variables were compared using the chi-square test of homogeneity or Fisher’s exact test. A p-value < 0.05 is considered statistically significant. ACS, acute coronary syndrome.

Parameters	Patients with acute coronary syndrome (ACS), Cases (n = 120)	Healthy participants, Controls (n = 40)	p-value	
Age (years)	55.6 ± 10.86	54.0 ± 8.93	0.399	
Male/Female (n/n)	107/13	34/6	0.573	
Smoker (n, %)	58 (48%)	6 (15%)	< 0.01	
Alcoholics (n, %)	61 (50%)	6 (15%)	< 0.01	
Presence of diabetes mellitus (n, %)	44 (37%)	1 (2.5%)	< 0.01	
Presence of hypertension (n, %)	50 (42%)	3 (7.5%)	< 0.01	
Presence of ischemic heart disease (n, %)	17 (14%)	0 (0%)	< 0.007	
Family history of diabetes mellitus (n, %)	32 (27%)	6 (15%)	0.133	
Family history of hypertension (n, %)	29 (24%)	5 (12.5%)	0.118	
Family history of ischemic heart disease (n, %)	18 (15%)	8 (20%)	0.458	
Time between onset of chest pain and collection (hours)	26.42 ± 7.23	-	-	
Type of ACS				
ST-segment elevation myocardial infarction (n, %)	82 (68%)	-	-	
Non-ST-segment elevation myocardial infarction (n, %)	37 (31%)	-	-	
Unstable angina (n, %)	1 (0.8%)	-	-	
Type of vessel disease (out of 59)				
Single-vessel disease (n, %)	17 (28%)	-	-	
Double-vessel disease (n, %)	21 (36%)	–	-	
Triple-vessel disease (n, %)	21 (36%)	-	-	
Person on active medication (n, %)	56 (47%)	4 (10%)	< 0.01	
Weight (kg)	73 (66–80)	78 (68-83)	0.032	
Height (m)	1.58 (1.54–1.62)	1.59 (1.55-1.63)	0.178	
Waist circumference (inch)	37 (35-40)	38 (36-42)	0.172	
Body mass index (kg/m^2^)	28.85 ± 3.75	29.92 ± 3.66	0.117	
Systolic blood pressure (mm Hg)	130 (118-140)	114 (110-117)	< 0.001	
Diastolic blood pressure (mm Hg)	80 (70-90)	74 (70-78)	< 0.001	
Pulse rate (beats/min)	84 (70-93)	84 (75-87)	0.867	
Hemoglobin (g/dL)	14.2 (13.4-14.8)	13.7 (12.8-14.6)	0.027	
White blood cells (thousands/mm^3^)	10.38 (8.93–12.63)	5.64 (4.89-7.37)	< 0.001	
Platelets (lakhs/mm^3^)	2.66 (2.19-3.15)	3.17 (2.41-4.01)	0.005	
Random blood glucose (mg/dL)	160 (127-209)	100 (74-149)	< 0.001	
Total cholesterol (mg/dL)	167 (140-202)	182 (159-204)	0.195	
Triglycerides (mg/dL)	117 (86-165)	145 (88-191)	0.324	
Low-density lipoprotein-cholesterol (mg/dL)	114 ± 33	106 ± 30	0.142	
Very low-density lipoprotein-cholesterol (mg/dL)	24 (17-36)	29 (20-38)	0.360	
High-density lipoprotein-cholesterol (mg/dL)	39 (33-46)	34 (28-38)	0.001	
Non-high-density lipoprotein-cholesterol (mg/dL)	128 (104–160)	144 (119-170)	0.041	
Urea (mg/dL)	24 (20-33)	18 (15-25)	< 0.001	
Creatinine (mg/dL)	0.90 (0.77-1.11)	0.82 (0.69-0.97)	0.015	
Creatine kinase-total (IU/L)	636 (452-1119)	74 (48-135)	< 0.001	
Creatine kinase-MB (IU/L)	54 (33-90)	5 (2-12)	< 0.001	

ACS patients (cases) and healthy participants (controls) were comparable with respect to age and gender. ACS patients had a significantly higher proportion of patients with smoking, alcohol drinking, HTN, ischemic heart disease (IHD), and DM than healthy participants (p < 0.01). However, no significant difference was observed in the family history of DM, HTN, and IHD between ACS patients and healthy participants. The mean duration between the onset of chest pain and the time of sample collection among the ACS patients was 26.42 ± 7.23 hours. The mean duration between the onset of chest pain and the time of sample collection among NSTEMI, STEMI, and UA patients was 27.90 ± 7.7, 25.70 ± 6.98, and 30 hours, respectively. Among the 120 patients with ACS, 82 (68%) patients were diagnosed with STEMI, 37 (31%) patients with NSTEMI, and one (0.8%) patient with UA. Coronary angiographic data were available only for 59 ACS patients, of whom 17 (28%) patients had SVD, 21 (36%) patients had DVD, and 21 (36%) patients had TVD. Out of 120 ACS patients, 56 (47%) patients were on active medications for DM, HTN, and IHD.

In terms of anthropometric measures, ACS patients had significantly lower body weight than controls (p = 0.032). No significant difference was seen in height, waist circumference, and BMI between ACS patients and healthy participants. ACS patients had significantly higher systolic blood pressure (SBP) and diastolic blood pressure (DBP) than healthy participants (p < 0.001). No significant difference was seen in pulse rate between the ACS patients and healthy participants. Hematological and biochemical parameters showed significantly lower levels of platelet count (p = 0.005) and significantly higher levels of hemoglobin (p = 0.027), WBC (p < 0.001), and random blood sugar (p < 0.001) among ACS patients than healthy participants. Furthermore, serum HDL-C (p = 0.001), creatinine (p = 0.015), and urea levels (p < 0.001) were significantly higher in ACS patients than in healthy participants, and significantly lower levels of non-HDL-C (p = 0.041) were found in ACS patients than in healthy participants. No significant difference was seen in serum total cholesterol, triglycerides, and very low-density lipoprotein cholesterol levels between ACS patients and healthy participants. Traditional markers for myocardial infarction, such as serum CK-total (p < 0.001) and CK-MB levels (p < 0.001), were significantly higher among ACS patients than in healthy participants.

Risk stratification was done using TIMI, GRACE, and Killip risk scores [13-15]. According to the TIMI scores, 97 (81%) patients were categorized under the low-risk group, 23 (19%) were categorized under the intermediate-risk group, and none were categorized under the high-risk TIMI group. In contrast, according to the GRACE score, 26 (22%) patients were categorized under the low-risk group, and 94 (78%) were categorized under the intermediate/high-risk group. According to the Killip score, 110 (92%) patients were categorized under Killip class 1, and 10 (8%) were categorized under Killip class 2-4.

Biomarker comparison

Significantly higher levels of serum H-FABP were observed in ACS patients when compared to those in healthy participants (2.619 (1.736-4.895) vs. 0.660 (0.419-2.608) ng/mL, p < 0.001) (Figure 1A). Also, significantly higher levels of serum NPQ were observed in ACS patients when compared to healthy participants (4.30 (2.29-5.60) vs. 1.30 (0.66-3.36) ng/mL, p < 0.001) (Figure 1B).

**Figure 1:**
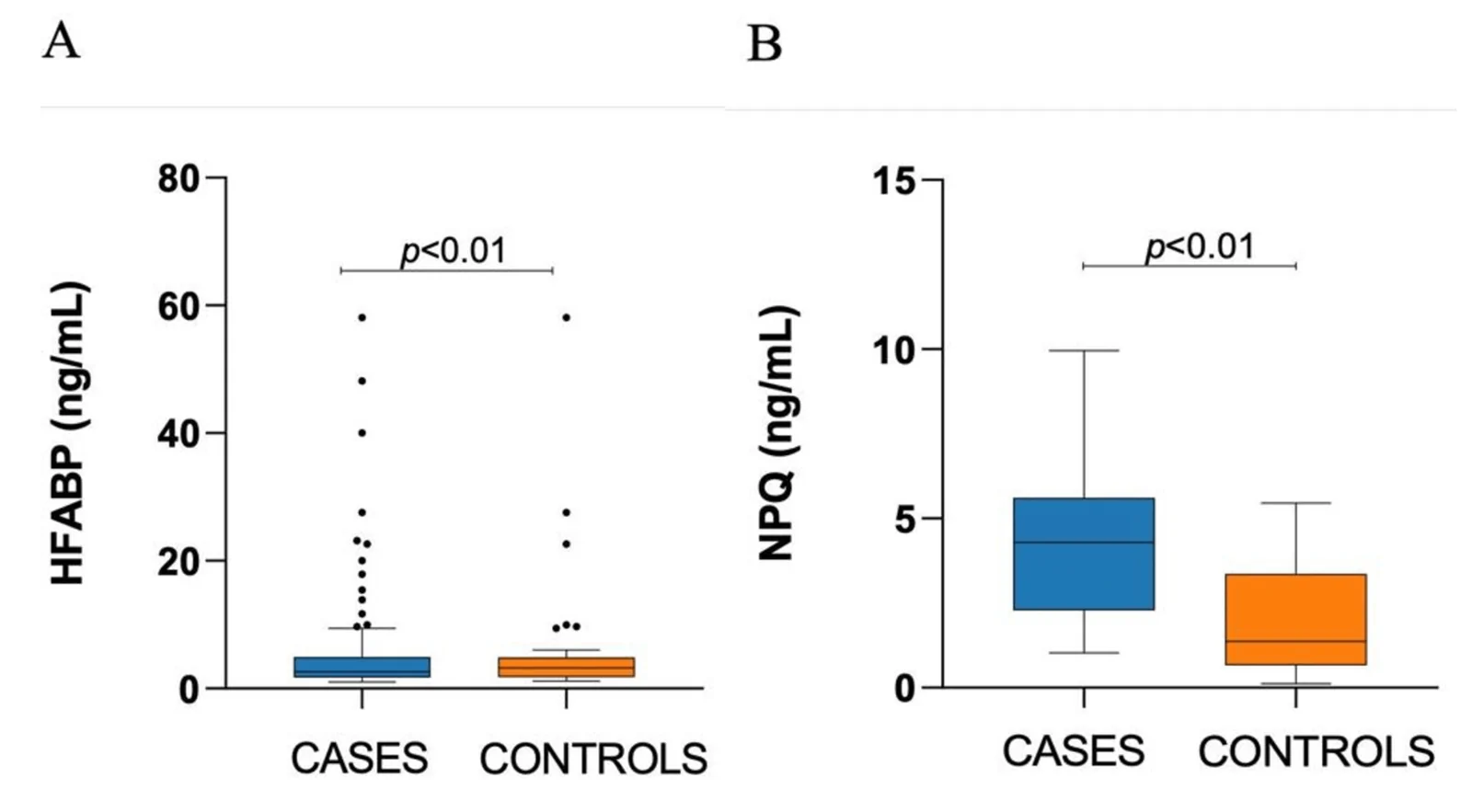
Comparison of serum (A) heart-type fatty acid-binding protein (H-FABP) and (B) neuropeptide Q (NPQ) levels between patients with acute coronary syndrome as cases and healthy participants as controls.

Serum NPQ levels significantly differ between the TIMI risk score groups (low- and intermediate-risk) (p = 0.029), as shown in Figure 2A. Also, serum NPQ levels do not significantly differ between the GRACE risk score groups (low- and intermediate/high-risk groups) (p = 0.246), as shown in Figure 2B. In addition, serum NPQ levels do not significantly differ between patients having SVD and DVD/TVD ACS (p = 0.684), as shown in Figure 2C. Also, serum NPQ levels do not significantly differ across Killip classes (Killip class 1 and class 2-4) (p = 0.55), as shown in Figure 2D.

**Figure 2:**
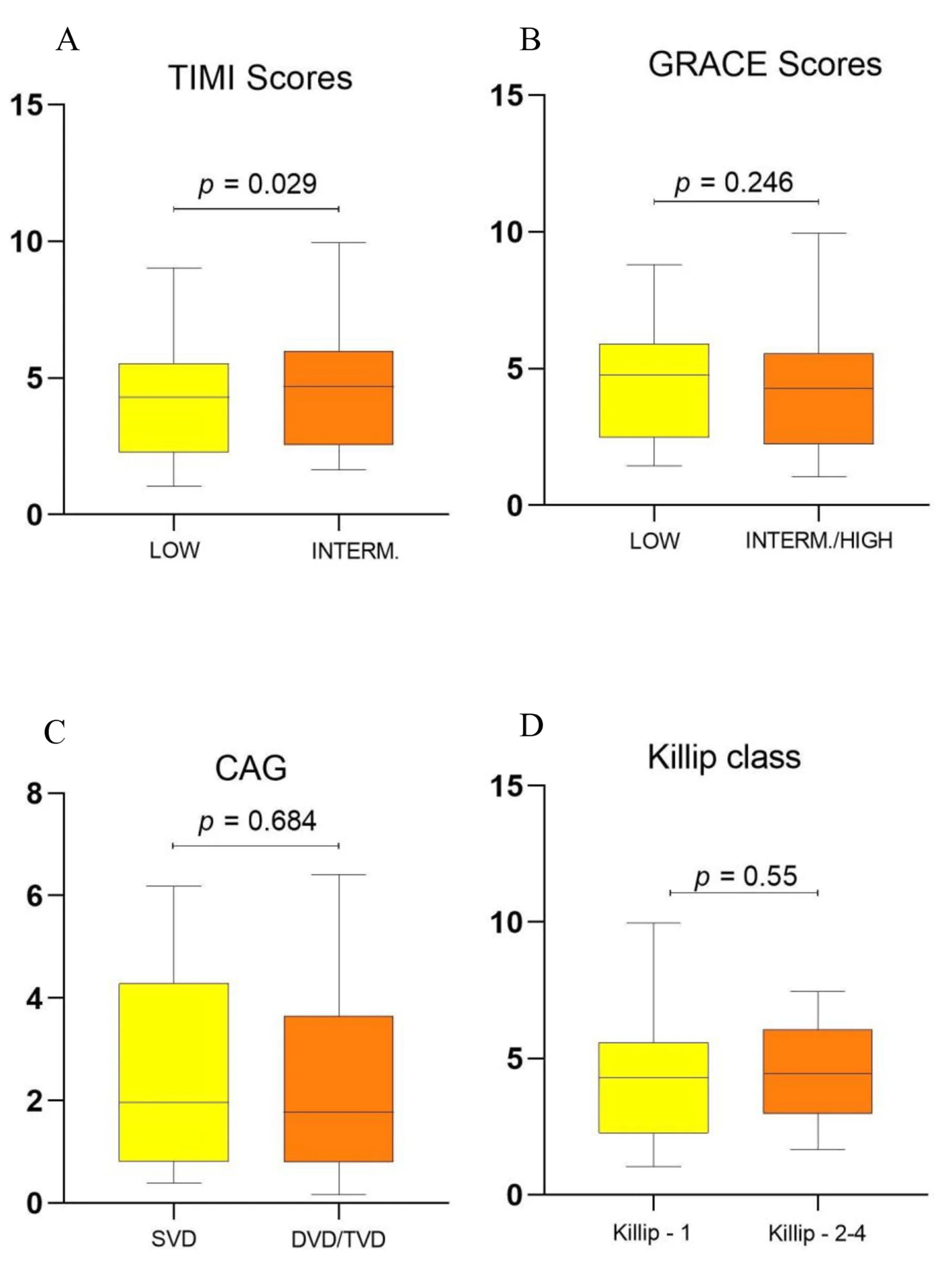
Comparison of serum neuropeptide Q (NPQ) levels (ng/mL) between (A) patients with low and intermediate Thrombolysis in Myocardial Infarction (TIMI) risk scores, (B) patients with low and intermediate/high Global Registry of Acute Coronary Events (GRACE) risks scores, (C) patients with single-vessel disease (SVD) and double-vessel disease (DVD)/triple-vessel disease (TVD) on coronary angiography (CAG), and (D) patients with Killip 1 and 2-4 classes.

Correlation analysis

In patients with ACS, serum NPQ levels showed a significant positive correlation with waist circumference (r = 0.507, p < 0.001) and body weight (r = 0.460, p < 0.001). NPQ showed a significant positive correlation with traditional markers of myocardial injury, such as CK-MB (r = 0.225, p = 0.013), CK-total (r = 0.233, p = 0.010), and H-FABP (r = 0.447, p < 0.001). Furthermore, NPQ also showed a significant negative correlation with WBC (r = -0.196, p = 0.032) and HDL-C (r = -0.437, p < 0.001), as shown in Table 2.

**Table 2:** Correlation of neuropeptide Q and heart-type fatty acid-binding protein levels with other variables among acute coronary syndrome patients (n = 120). All r-values reported are based on Pearson or Spearman’s correlation tests. A p-value < 0.05 is considered statistically significant. H-FABP, heart-type fatty-acid binding protein; NPQ, neuropeptide Q.

Parameters	Heart-type fatty acid-binding protein (H-FABP)		Neuropeptide Q (NPQ)	
	r	p-value	r	p-value
Age	–0.071	0.438	–0.102	0.268
Gender	–0.106	0.251	–0.112	0.224
Presence of diabetes	–0.013	0.891	0.014	0.882
Presence of hypertension	–0.096	0.296	–0.020	0.825
Presence of ischemic heart disease	–0.096	0.296	–0.020	0.825
Onset of chest pain	0.014	0.883	0.040	0.664
Weight	0.643	< 0.001	0.460	< 0.001
Height	0.011	0.904	–0.045	0.625
Waist circumference	0.458	< 0.001	0.507	< 0.001
Body mass index	0.011	0.906	0.076	0.410
Systolic blood pressure	0.098	0.288	0.004	0.963
Diastolic blood pressure	0.018	0.848	–0.053	0.562
Pulse rate	0.055	0.550	–0.068	0.459
Hemoglobin	–0.111	0.227	–0.019	0.838
White blood cell	–0.133	0.148	–0.196	0.032
Platelets	–0.121	0.188	–0.036	0.699
Random blood glucose	0.098	0.288	–0.067	0.468
Total cholesterol	–0.023	0.801	–0.023	0.802
Triglycerides	–0.142	0.123	–0.106	0.248
High-density lipoprotein-cholesterol	–0.292	0.001	–0.437	< 0.001
Non-high-density lipoprotein-cholesterol	0.005	0.960	0.009	0.925
Very low-density lipoprotein-cholesterol	–0.095	0.303	–0.081	0.379
Low-density lipoprotein-cholesterol	–0.020	0.825	0.004	0.964
Urea	–0.002	0.985	0.109	0.236
Creatinine	0.062	0.500	0.091	0.322
Creatine kinase-total	0.359	< 0.001	0.233	0.010
Creatine kinase-MB	0.335	< 0.001	0.225	0.013
Thrombolysis in Myocardial Infarction (TIMI) score	0.070	0.449	–0.089	0.334
Global Registry of Acute Coronary Events (GRACE) score	–0.021	0.818	–0.163	0.075
Killip scores	–0.015	0.875	–0.038	0.682
NPQ	0.447	< 0.001	1.000	–
H-FABP	1.000	–	0.447	< 0.001

Linear regression analysis

Assuming serum H-FABP levels as one of the indicators of myocardial necrosis, the association between serum NPQ and log H-FABP levels was studied by linear regression analysis (multivariate) after adjusting for significant confounding parameters like weight, waist circumference, WBC, and serum HDL-C levels. The findings indicated that serum NPQ level was associated with log H-FABP in patients with a low-risk TIMI score (β = 0.349, p = 0.001), while weight, waist circumference, WBC, and HDL-C were not significant. The final model was statistically significant (adjusted R² = 0.085, p = 0.022). However, in patients with intermediate TIMI risk, none of the variables, including NPQ, were significantly associated with log H-FABP, and the overall model was not statistically significant (adjusted R² = 0.040, p = 0.358). In patients with low-risk GRACE, we found that NPQ was significantly associated with log H-FABP (β = 0.650, p = 0.002), while other variables were not significant. The overall model was statistically significant (adjusted R² = 0.297, p = 0.031), as shown in Table 3. Conversely, in the intermediate/high-risk group, none of the variables, including NPQ, were significantly associated, and the overall model was not statistically significant (p = 0.954). Because the model performed worse than a baseline intercept alone (adjusted R² = -0.082), it holds no explanatory value. Within the Killip class 1 group, multiple linear regression revealed that NPQ was associated with log H-FABP level (β = 0.385, p < 0.001). The overall regression model was statistically significant (adjusted R² = 0.118, p = 0.003), as shown in Table 3. However, in the Killip class 2-4 group, none of the variables, including NPQ, were statistically significant, and the overall model was not statistically significant (adjusted R² = -0.055, p = 0.553). In the SVD group, multiple linear regression indicated that none of the variables, including NPQ, were statistically significantly associated with log H-FABP levels, and the overall model was not statistically significant (adjusted R² = 0.171, p = 0.224), as shown in Table 3. Similarly, in the DVD/ TVD groups, none of the variables, including NPQ, were associated with log H-FABP levels, and the overall model was not statistically significant (adjusted R² = 0.029, p = 0.308).

**Table 3:** Multiple linear regression analysis of serum log heart-type fatty acid-binding protein levels in various groups. The regression coefficient (b) was derived from a multiple linear regression model. A p-value < 0.05 was considered statistically significant. VIF, variance inflation factor; WBC, white blood cells; HDL-C, high-density lipoprotein cholesterol; NPQ, neuropeptide Q; GRACE, Global Registry of Acute Coronary Events; SVD, single-vessel disease.

Variables	Patients with Killip class 1				
	b	t	p-value	Tolerance	VIF
Weight (kg)	0.073	0.680	0.498	0.708	1.412
Waist circumference (inch)	–0.054	–0.503	0.616	0.693	1.444
White blood cell (thousands/mm^3^)	–0.069	–0.761	0.448	0.997	1.003
High-density lipoprotein-cholesterol (mg/dL)	–0.014	–0.154	0.878	0.973	1.028
Neuropeptide Q (ng/mL)	0.385	4.280	< 0.001	0.998	1.002
Variables	Patients with low-risk GRACE score				
	b	t	p-value	Tolerance	VIF
Weight (kg)	0.033	0.163	0.872	0.666	1.502
Waist circumference (inch)	0.151	0.707	0.488	0.617	1.622
White blood cell (thousands/mm^3^)	0.107	0.599	0.556	0.879	1.138
High-density lipoprotein-cholesterol (mg/dL)	0.025	0.133	0.896	0.816	1.226
Neuropeptide Q (ng/mL)	0.650	3.561	0.002	0.843	1.186
Variables	Patients with SVD				
	b	t	p-value	Tolerance	VIF
Weight (kg)	–0.268	–1.023	0.328	0.757	1.320
Waist circumference (inch)	0.577	2.180	0.052	0.740	1.351
White blood cell (thousands/mm^3^)	–0.107	–0.448	0.663	0.902	1.108
High-density lipoprotein-cholesterol (mg/dL)	–0.054	–0.227	0.825	0.905	1.105
Neuropeptide Q (ng/mL)	0.257	1.099	0.295	0.950	1.053

Logistic regression analysis of factors correlated with ACS

Upon logistic regression analysis (univariate), it was observed that body weight (OR: 0.952, 95% CI: 0.911-0.994, p = 0.025), WBC (OR: 1.728, 95% CI: 1.427-2.093, p < 0.001), HDL-C (OR: 1.069, 95% CI: 1.023-1.117, p = 0.003), and NPQ (OR: 1.961, 95% CI: 1.502-2.561, p < 0.001) levels were substantially linked with ACS among the study participants, while waist circumference was not statistically significant (p = 0.081). In logistic regression analysis (multivariate), after adjusting for potential confounding variables, only WBC (adjusted OR = 1.694, 95% CI: 1.392-2.061, p < 0.001) and NPQ (adjusted OR = 1.981, 95% CI: 1.465-2.782, p < 0.001) were independently linked with ACS among the study participants, as shown in Table 4.

**Table 4:** Univariate and multivariate logistic regression analysis of factors associated with acute coronary syndrome (ACS) outcome in the study participants. The regression coefficient (b) with a 95% confidence interval was derived from univariate and multivariate regression models, with ACS as the dependent variable. Candidate variables are weight, waist circumference, white blood cells, high-density lipoprotein cholesterol, and neuropeptide Q. Retained variables are white blood cells and neuropeptide Q. A two-step selection strategy was used. Variables significant on univariate analysis were considered. A purposeful entry rule was applied, retaining only variables with a final independent association threshold of p < 0.05. CI, confidence interval.

Variables	Univariate		Multivariate	
	b (95% CI)	p-value	b (95% CI)	p-value
Weight (kg)	0.952 (0.911–0.994)	0.025	–	–
Waist circumference (inch)	0.929 (0.854–1.009)	0.081	–	–
White blood cell (thousands/mm^3^)	1.728 (1.427–2.093)	< 0.001	1.694 (1.392–2.061)	< 0.001
High-density lipoprotein-cholesterol (mg/dL)	1.069 (1.023–1.117)	0.003	–	–
Neuropeptide Q (ng/mL)	1.961 (1.502–2.561)	< 0.001	1.981 (1.465–2.782)	< 0.001

ROC curve analysis of serum NPQ and H-FABP levels to predict ACS

ROC curve analysis revealed that the optimum cut-off value for serum NPQ levels was 2.53 ng/mL in predicting ACS with 71% sensitivity and 67.5% specificity, LR+ of 2.18 (95% CI: 1.37-3.46), LR- of 0.43 (95% CI: 0.30-0.61) and the AUC of 0.808 (95% CI: 0.731-0.886, p < 0.01). Similarly, the cut-off value for predicting ACS using serum H-FABP was 1.92 ng/mL with 70% sensitivity and a specificity of 72.5%, LR+ of 2.55 (95% CI: 1.52-4.27), LR- of 0.41 (95% CI: 0.30-0.58) and with the AUC of 0.797 (95% CI: 0.692-0.901, p < 0.001), as shown in Figure 3.

**Figure 3:**
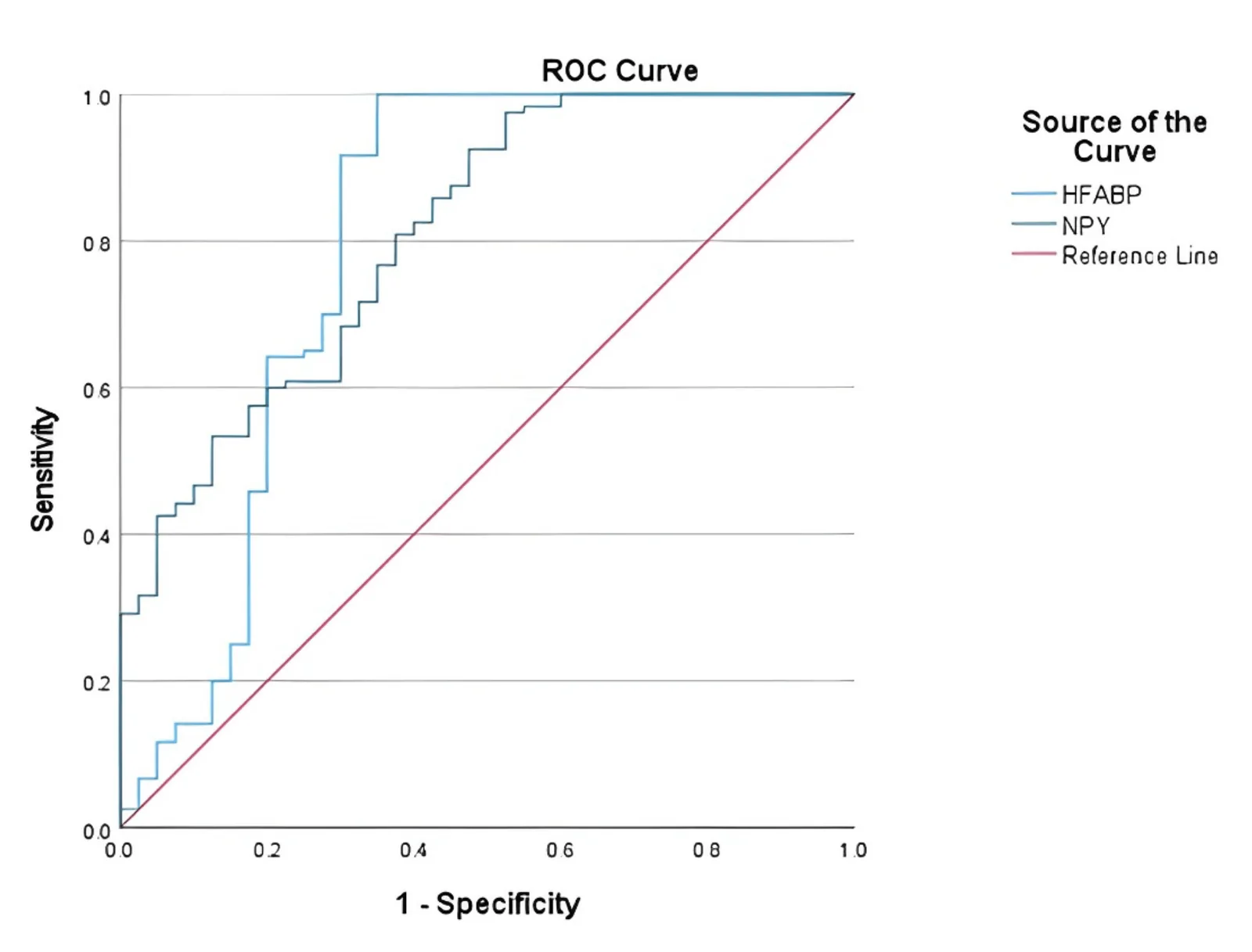
Receiver operating characteristic curve analysis of serum neuropeptide Q (NPQ) and heart-type fatty acid-binding protein (H-FABP) levels to predict acute coronary syndrome in the study participants.

## Discussion

Considering the worldwide increase in the incidence and mortality of ACS patients and the constraints of conventional cardiac markers in ACS diagnosis, our study aimed to characterize the utility of serum NPQ levels in patients with ACS. Our study revealed that serum NPQ levels may be considered as one of the biomarkers for ACS diagnosis and prognosis.

NPQ is a peptide hormone expressed in the central and peripheral tissues, including the heart [19]. It plays an important role in metabolic regulation and cardioprotection, especially protecting cardiomyocytes from hypoxia-induced mitochondrial dysfunction [11]. The study consists of 120 ACS patients as cases and 40 healthy participants as controls to characterize the utility of serum NPQ levels in ACS patients. Our study showed that cases had a higher prevalence of lifestyle and metabolic risk factors, like alcohol consumption, smoking, HTN, and DM, than controls, leading to ACS precipitation; however, the remaining risk factors, like obesity, gender, age, and family history of DM, HTN, and IHD, may not contribute to ACS. The high proportion of STEMI patients compared with those with NSTEMI and UA among cases may reflect a greater atherosclerotic burden and its complications, as well as early referral to tertiary care centers for prompt revascularization.

Our study found higher serum NPQ levels in patients with ACS compared to healthy participants. This finding observed in our study contradicts the findings from earlier studies; for example, Çiftçi et al. showed that serum NPQ levels were significantly lower in 100 acute myocardial infarction (AMI) patients when compared to 50 control subjects presenting with non-cardiac chest pain [17]. This contradiction may be due to population differences, metabolic influences, or the time of sample collection. The proposed explanation for elevated serum NPQ levels in our study is due to the rapid release of NPQ from the damaged cardiomyocytes into the circulation or compensatory release of NPQ to protect the cardiomyocyte mitochondria from hypoxia-induced dysfunction [11,20]. Released NPQ acts as a ligand and binds to the galanin receptor 2 (GALR2) and galanin receptor (GALR3), resulting in activation of fatty acid metabolism by promoting expression of carnitine palmitoyl transferase 1 (CPT1), cluster of differentiation 36 (CD36), peroxisome proliferator-activated receptor alpha (PPAR-α), acyl-CoA dehydrogenase medium chain (ACADM), peroxisome proliferator-activated receptor gamma coactivator 1 alpha (PGC1-α), transcription factor A, mitochondrial (TFAM), and the respiratory chain complex [11].

Interestingly, our data showed a significant positive correlation between NPQ and anthropometric markers, such as weight and waist circumference, among patients with ACS. These findings contradict earlier studies, which reported obesity-dependent downregulation of NPQ levels, in which increased BMI and general obesity in various animal and human models were generally associated with lower circulating levels of NPQ [10,21-23]. Our proposed explanation for this contradiction may be due to the acute pathological state associated with ACS; it may alter the normal regulatory state of NPQ and lead to elevated NPQ levels [24]. In our present study, a significant negative correlation was observed between serum NPQ and HDL-C levels. While previous experiments have suggested that NPQ plays a critical role in lipid metabolism, it acts mainly by reducing triglycerides and low-density lipoprotein cholesterol and increasing HDL-C in various animal models of metabolic disorders [25-27]. Surprisingly, our study revealed that serum HDL-C levels were higher among cases compared to controls. Though HDL-C is traditionally considered protective, recent studies have highlighted that elevated HDL-C does not always confer cardiovascular protection, a phenomenon called the "HDL paradox" [28]. Furthermore, various experimental studies have proved that NPQ may act as a central modulator of cardiovascular function, influencing parameters like mean arterial pressure and heart rate [27,29]. In contrast, our study did not demonstrate any significant correlation between NPQ levels, SBP, and DBP; this lack of association may be explained by extensive use of antihypertensive drugs among the patients with ACS.

Additionally, our study shows a significant negative correlation between serum NPQ levels and WBC, suggesting a potential association with inflammation in ACS. Inflammation plays an important role in the pathogenesis of ACS, especially in plaque destabilization and rupture. Previous studies indicate that NPQ may have anti-inflammatory properties, such as suppression of pro-inflammatory cytokines like tumor necrosis factor-alpha, interleukin-6, and high-sensitivity C-reactive protein in various animal models [25,30]. The inverse relationship observed between serum NPQ levels and WBC count in our study may indicate a protective or adaptive response. Also, in our present study, serum NPQ levels were significantly associated with conventional markers of myocardial infarction, like serum CK-MB, CK-total, and H-FABP levels, among ACS patients. H-FABP is a protein located predominantly in the cytoplasm of cardiomyocytes, and it plays a critical role in transporting fatty acids for their metabolism and also promotes their metabolism. Because of this specificity and its circulatory dynamics, it is considered one of the biomarkers of myocardial injury and is helpful for early detection and prognosis of AMI [31]. We understand that our mean onset-to-sampling time of 26.42 hours falls outside the upward trajectory period where H-FABP serves as a primary diagnostic tool. At this sub-acute stage, H-FABP is largely in the clearing phase from circulation. Therefore, H-FABP is considered a retrospective surrogate of initial myocardial injury severity rather than an early diagnostic marker. Consequently, the significant correlation between serum NPQ and H-FABP levels suggests that serum NPQ levels could be considered one of the markers for ACS. Therefore, serum NPQ levels might be used to identify high-risk individuals who have not undergone angiography, to be transitioned from medical treatment to invasive management.

To further evaluate this relationship, multivariate linear regression was performed to assess the association between serum NPQ and log-transformed H-FABP levels, after adjusting for potential confounders, including weight, waist circumference, WBC, and HDL-C across different subgroups. It showed that NPQ was associated with log H-FABP levels in patients classified as low-risk according to TIMI, GRACE, and Killip scores, with a statistically significant regression model. In contrast, in intermediate-risk TIMI and intermediate/high-risk GRACE, and Killip class 2-4, NPQ was not significantly associated with log H-FABP levels, and the regression model was also not significant. For a biologically plausible marker of acute myocardial injury, one expects an accentuating risk gradient as baseline clinical risk increases (e.g., across increasing TIMI or GRACE scores). The complete absence of an effect in higher-risk cohorts strongly suggests that the low-risk finding is not a true pathophysiological signal, but rather an artefact of residual confounding, selection bias, or statistical noise stemming from low absolute event rates in that stratum. In higher-risk ACS patients, where true ischemic and necrotic events dominate the clinical picture, the marker loses all predictive utility.

In summary, while the current literature largely describes NPQ as a peptide depleted in chronic metabolic diseases, the present study demonstrated a significant elevation of serum NPQ levels in ACS. The strong positive correlation with H-FABP and CK-MB suggests that NPQ is released in response to myocardial injury. These findings align with the hypothesis that NPQ plays an important role in cardioprotection and highlight its utility as a biomarker in patients with ACS.

This study has a few limitations. First, as it was a case-control study, it was challenging to assess the cause-and-effect link between serum NPQ and ACS. Serum NPQ levels were compared between ACS patients and healthy participants, rather than patients with chest pain. H-FABP was used as a surrogate marker for myocardial injury, as it was measured around 26 hours after onset of chest pain. The NPQ threshold was derived and evaluated in a similar ACS cohort without optimism correction. The sample size of 120 ACS patients and 40 controls was adequate for a pilot study; it was relatively small, particularly after stratification into clinical subgroups, which resulted in reduced statistical power and limited the stability of regression models. Because of the single-center design, it may introduce potential demographic and selection bias. Also, due to the absence of long-term follow-up, we were unable to assess the prognostic utility of NPQ in patients with ACS. Coronary angiography reports were available for a limited number of ACS patients. In addition, the limited availability of existing literature on NPQ in AMI restricts direct comparison and external validation of our present findings.

## Conclusions

NPQ exhibited a sharp elevation in ACS patients, contrasting with findings from prior human studies and chronic metabolic disease depletion patterns. This unexpected increase points to an acute release mechanism during cardiac events rather than a stable diagnostic role, while same-sample threshold discrimination remains insufficient to establish clinical utility. Future investigations require evaluation within a large, independent validation cohort utilizing a pre-set cut-off value.
